# Non-Alcoholic Fatty Liver Disease and Risk of Macro- and Microvascular Complications in Patients with Type 2 Diabetes

**DOI:** 10.3390/jcm11040968

**Published:** 2022-02-12

**Authors:** Alessandro Mantovani, Andrea Dalbeni, Giorgia Beatrice, Davide Cappelli, Fernando Gomez-Peralta

**Affiliations:** 1 Section of Endocrinology, Diabetes and Metabolism, Department of Medicine, University and Azienda Ospedaliera Universitaria Integrata of Verona, 37126 Verona, Italy; giorgiabeatricejb@gmail.com (G.B.); davide.cappelli3@gmail.com (D.C.); 2 Section of General Medicine C and Liver Unit, University and Azienda Ospedaliera Universitaria Integrata of Verona, 37126 Verona, Italy; andrea.dalbeni@aovr.veneto.it; 3 Endocrinology and Nutrition Unit, Segovia General Hospital, 40002 Segovia, Spain

**Keywords:** non-alcoholic fatty liver disease, NAFLD, non-alcoholic steatohepatitis, NASH, type 2 diabetes, cardiovascular disease, cardiovascular complications, CVD

## Abstract

Non-alcoholic fatty liver disease (NAFLD) is considered the hepatic manifestation of metabolic syndrome. To date, NAFLD is the most frequent chronic liver disease seen day by day in clinical practice across most high-income countries, affecting nearly 25–30% of adults in the general population and up to 70% of patients with T2DM. Over the last few decades, it clearly emerged that NAFLD is a “multisystemic disease” and that the leading cause of death among patients with NAFLD is cardiovascular disease (CVD). Indeed, several observational studies and some meta-analyses have documented that NAFLD, especially its advanced forms, is strongly associated with fatal and non-fatal cardiovascular events, as well as with specific cardiac complications, including sub-clinical myocardial alteration and dysfunction, heart valve diseases and cardiac arrhythmias. Importantly, across various studies, these associations remained significant after adjustment for established cardiovascular risk factors and other confounders. Additionally, several observational studies and some meta-analyses have also reported that NAFLD is independently associated with specific microvascular conditions, such as chronic kidney disease and distal or autonomic neuropathy. Conversely, data regarding a potential association between NAFLD and retinopathy are scarce and often conflicting. This narrative review will describe the current evidence about the association between NAFLD and the risk of macro- and microvascular manifestations of CVD, especially in patients with T2DM. We will also briefly discuss the biological mechanisms underpinning the association between NAFLD and its advanced forms and macro- and microvascular CVD.

## 1. Introduction

**Figure 1:**
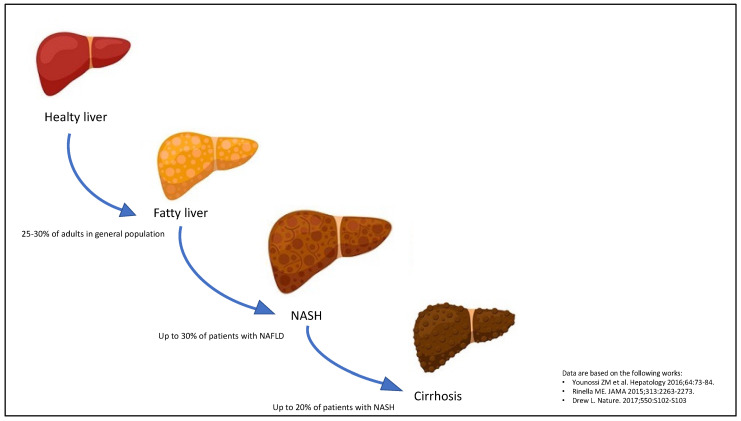
Progression of non-alcoholic fatty liver disease (NAFLD). The stages of NAFLD development classically are simple steatosis, non-alcoholic steatohepatitis (NASH) and cirrhosis.

Non-alcoholic fatty liver disease (NAFLD) is a metabolic liver disease, which classically includes a spectrum of progressive pathological conditions, ranging from simple steatosis to non-alcoholic steatohepatitis (NASH) with different grades of fibrosis and cirrhosis (Figure 1) [1,2]. At present, NAFLD is the most common chronic liver disease seen day by day in clinical practice, as it affects roughly 25–30% of adults in the general population across various high-income countries [3], up to 70% of patients with type 2 diabetes (T2DM) [4] and all patients with obesity [5]. On the other side, most NAFLD patients have relevant metabolic comorbidities, including atherogenic dyslipidemia (~70%), obesity (~50%), hypertension (~40%) and T2DM (~30%) [6]. In this regard, alongside the increasing prevalence of metabolic syndrome worldwide, the overall prevalence of NAFLD is believed to rise further in the coming years.

The diagnosis of NAFLD is a diagnosis of exclusion [7]. It is essentially based on the following criteria: (a) presence of hepatic steatosis, as detected by specific serum biomarker scores (e.g., fatty liver index [FLI]), imaging techniques or liver histology, (b) no alcohol consumption (<20 g/day for women and <30 g/day for men), and (c) no other secondary causes of liver steatosis (e.g., virus, hepatotoxic drugs, hemochromatosis, autoimmune hepatitis) [7]. In the last two years, several experts in the field and many scientific societies have proposed a revision of the terminology, switching from NAFLD to metabolic-associated fatty liver disease (MAFLD) [8,9]. In this regard, the diagnosis of MAFLD can be undertaken from the presence of hepatic steatosis and at least one of the following criteria: (a) overweight/obesity, (b) T2DM, and (c) metabolic dysregulation (i.e., two or more factors among increased waist circumference, hypertriglyceridemia, low serum HDL-cholesterol levels, hypertension, impaired fasting glucose, insulin resistance and chronic inflammation) [8,9]. Several studies and some meta-analyses have recently indicated that the MAFLD criteria can identify more individuals with liver damage than NAFLD criteria [10]. However, given that there is still an intense debate about which term should be used [11,12], we have preferred to use still NAFLD term in this manuscript.

Importantly, in the last decades, it has also become clear that NAFLD is a “multisystemic” disease [13]. Indeed, several observational studies and some meta-analyses have clearly documented that NAFLD is independently associated with serious hepatic complications (e.g., hepatic decompensation, hepatocellular carcinoma [HCC]) [5], but also with an increased risk of developing cardiovascular disease (CVD) [14], T2DM [15], chronic kidney disease (CKD) [16] and some extra-hepatic cancers [17]. Notably, among the various hepatic and extra-hepatic complications related to NAFLD, CVD is the leading cause of death among NAFLD patients.

**Figure 2:**
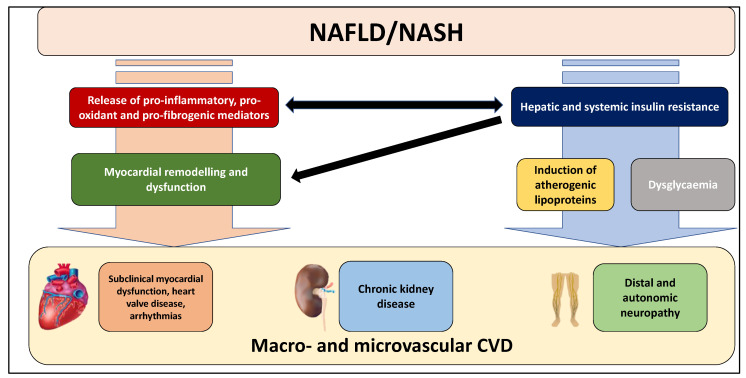
Macro- and microvascular manifestations of cardiovascular disease (CVD) linked to NAFLD and its advanced forms. Several observational studies and meta-analyses have clearly reported that NAFLD, mainly in its advanced forms, is strongly associated with an increased risk of sub-clinical myocardial remodelling and dysfunction, heart valve diseases, cardiac arrhythmias, chronic kidney disease, and distal or autonomic neuropathy. See text for details.

This narrative review will discuss the current evidence regarding the association between NAFLD and the risk of macro- and microvascular CVD (Figure 2). In particular, it will describe the association between NAFLD and the risk of sub-clinical myocardial remodelling and dysfunction, heart valve diseases, cardiac arrhythmias, chronic kidney disease, distal or autonomic neuropathy, retinopathy and fatal and non-fatal cardiovascular events. A brief insight into the biological mechanisms underpinning the association between NAFLD and macro- and microvascular complications has been also given.

## 2. Biological Link between Non-Alcoholic Fatty Liver Disease (NAFLD) and Cardiovascular Disease (CVD)

The underlying biological mechanisms responsible for the association between NAFLD and the risk of specific cardiac complications are not completely established to date. It is beyond the scope of this narrative review to illustrate in detail the current evidence suggesting a specific role of NAFLD in the development and progression of various cardiac complications. That said, in brief, accumulating evidence now indicates that NAFLD, especially its severe forms, may play a part in the pathophysiology of cardiac complications through different mechanisms, such as:

(a) hepatic lipid accumulation (e.g., di-acyl glycerol [DAG]) in NAFLD patients impairs insulin signalling, thereby conditioning insulin resistance (IR) through different mechanisms, including the inhibition of phosphorylation of insulin receptor substrate-1 (IRS-1) [18] and the activation of protein kinase C (PKC)-e that can inhibit the action of insulin receptor and promote the lipid accumulation [19]. In particular, hepatic and systemic insulin resistance is one of the primary mechanisms for inducing atherogenic lipoproteins and dysglycaemia. Notably, both atherogenic dyslipidemia and dysglycaemia mediate CVD risk in NAFLD patients with T2DM;
(b) the release into the bloodstream of several pro-inflammatory (e.g., tumour necrosis factor-a [TNF-a], interleukin-6 [IL-6]), pro-oxidant and pro-coagulant factors (e.g., fibrinogen, factor VIII, plasminogen activator inhibitor-1) as well as pro-fibrogenic mediators. In particular, the synthesis of lipids, including DAG, may also contribute to the hepatic production of inflammatory cytokines and pro-coagulant factors [13,20,21,22];
(c) the bidirectional relationship between NAFLD and hypertension [23]. Several observational studies and some meta-analyses have reported that patients with NAFLD have an increased risk of developing hypertension [24], thus suggesting that this association may partly mediate the relationship between NAFLD and cardiac complications and that that NAFLD may be a consequence, but also a cause of hypertension [23];
(d) patients with NAFLD have early changes in myocardial substrate metabolism inducing cardiac functional disturbances, probably conditioning a higher risk of heart failure [25] and arrhythmias [22,26];
(e) chronic hyperglycemia induces an inflammatory and osteoblastic phenotype in valvular interstitial cells in experimental models of aortic valve sclerosis [27]. Increased valvular inflammation, through a systemic inflammatory state, could also mediate the increased cardiac valve sclerosis in NAFLD patients, independent of the presence of T2DM;
(f) experimental data also indicate that NAFLD, mainly when advanced stages occur, may contribute to the activation of multiple pathways involved in the pathophysiology of CKD [10,28]. In this regard, atherogenic dyslipidaemia, hypertension, insulin resistance, oxidative stress and pro-inflammatory factors that, as mentioned above, are promoted and exacerbated by NAFLD status, may directly contribute to the vascular and renal damage [28]. Moreover, impaired activation of the renin-angiotensin system (RAS) may also contribute to the renovascular injury by inflammation pathways [28]. Finally, accumulating evidence also suggests a potential and independent association between *PNPLA3* (patatin like phospholipase domain containing-3) rs738409, which is the most important polymorphism associated with NAFLD and its advanced forms [29], and kidney dysfunction [28].

All these factors can promote myocardial remodelling and dysfunction, thereby predisposing to the development of various cardiac complications [13,20,21,22].

## 3. Risk of Microvascular Complications

### 3.1. Chronic Kidney Disease (CKD)

Several observational studies and some meta-analyses have reported that NAFLD, as detected by indirect biomarkers of steatosis, ultrasonography or liver biopsy, is associated with an increased risk of prevalent and incident chronic kidney disease (CKD) in patients with and without T2DM, independent of established cardio-metabolic risk factors, diabetes-related variables and other potential confounders [28,30]. In a recent 2022 meta-analysis of 13 longitudinal studies for a total of 1,222,032 patients (~28% with NAFLD as detected by biomarkers, International Classification of Diseases [ICD] codes, imaging techniques or biopsy) and 33,840 new cases of incident CKD stage (defined as CKD stage ≥3 and/or overt proteinuria) over a median follow-up of nearly 10 years, our research group reported that NAFLD was associated with a 43% increased risk of incident CKD (random-effects hazard ratio 1.43, 95% confidence interval 1.33 to 1.54; I^2^ = 60.7%), independent of age, sex, obesity, hypertension, T2DM and other CKD risk factors [16]. In a 2018 meta-analysis, the same research group documented that such association was slightly higher when the analysis was restricted to cohort studies involving exclusively patients with diabetes mellitus (random-effects hazard ratio 1.56, 95% confidence interval 1.07–2.05; I^2^ = 0%) [31]. Interestingly, accumulating observational studies using vibration controlled transient elastography (VCTE), as non-invasive method to evaluate the degree of liver fibrosis, also reported an independent association between liver stiffness and renal dysfunction. In this regard, for instance, in a 2022 systematic review and meta-analysis of seven cross-sectional studies for a total of 7736 individuals with NAFLD, Ciarduillo et al. showed that liver fibrosis (as assessed by VCTE) was associated with an increased risk of prevalent CKD (defined as eGFR < 60 mL/min/1.73 m^2^ and urinary albumin to creatinine ratio ≥30 mg/g) (random-effects odds ratio 2.49, 95% confidence interval 1.89–3.29; I^2^ = 46.5%), as well as with an increased risk of prevalent albuminuria (random-effects odds ratio 1.98, 95% confidence interval 1.29–3.05; I^2^ = 46.5%) [32]. However, it should be noted that, at present, only few observational studies on this topic have used liver biopsy for the diagnosis of NAFLD, which is the reference standard for diagnosing and staging NAFLD [1,2]. Conversely, most available studies on this topic have used liver ultrasonography, which is to date the recommended first-line imaging method for detecting NAFLD in clinical practice [1,2], able to accurately detect mild-to-moderate hepatic steatosis, as assessed by liver biopsy [7].

Notably, the presence of NAFLD may be even associated with CKD progression [33]. In a cohort study of nearly 1500 CKD patients who underwent periodic health check-ups, Jang et al. showed that age- and sex-adjusted decline in eGFR values was higher in patients with NAFLD (as detected by ultrasonography) when compared with those without NAFLD [34]. In that study, interestingly, the decline in estimated eGFR related to NAFLD was even higher in patients with higher NAFLD fibrosis score (which is an indirect marker of advanced liver fibrosis), in those with proteinuria and/or low eGFR values at baseline and in those who were active smokers or had hypertension at baseline [34]. Although additional studies are needed, preliminary evidence also indicates that the improvement in liver histology in NAFLD patients is associated with improved kidney function [33,35].

Observational studies involving patients with and without T2DM have reported that the presence of the G allele of rs738409 in the *PNPLA3* gene is associated with lower eGFR values and/or higher prevalence of CKD, even after adjustment for the presence of NAFLD and other cardio-renal risk factors [28,30,33,36,37,38,39]. In a cross-sectional study including 157 Italian patients with T2DM, who underwent liver ultrasonography and kidney function assessment, our research group reported that the presence of the G allele of rs738409 in the *PNPLA3* gene was associated with an increased risk of CKD (defined as <60 mL/min/1.73 m^2^ and/or abnormal albuminuria), independent of liver disease severity, cardiorenal risk factors and other potential confounders [37]. Interestingly and notably, in that study, the authors also found that PNPLA3 mRNA expression was greatest in the liver and renal cortex, thereby suggesting that the *PNPLA3* rs738409 variant might contribute, at least in part, to the impaired kidney function in these patients [37]. These findings have also been confirmed in some cohorts of children and adolescents [40,41,42].

Taken together, these data strongly indicate that patients with NAFLD, especially those with severe forms, have an increased risk of developing CKD, independent of several cardio-renal risk factors and other confounders [28,33]. Interestingly, novel data also suggest that MAFLD criteria might identify patients with CKD better than NAFLD criteria [43]. However, seeing the observational nature of all studies available so far, it is essential to underline that a causal relationship between NAFLD and incident CKD cannot be proven yet [28,33].

### 3.2. Distal Symmetric Polyneuropathy and Autonomic Neuropathy

Some observational studies [44,45,46], although not all [47,48], have documented an association between NAFLD and the risk of prevalent distal symmetric polyneuropathy in T2DM patients, independent of multiple cardio-metabolic risk factors and diabetes-related variables. In a cross-sectional study involving roughly 400 outpatients with T2DM attending five Italian diabetes centers, who underwent liver ultrasonography, vibration controlled transient elastography (by FibroScan^®^) and evaluation of microvascular diabetic complications, Lombardi et al. documented that significant liver fibrosis (i.e., liver stiffness measurement [LSM] ≥ 7.0 and 6.2 kPa with M and XL probes, respectively) was independently associated with higher prevalence of microvascular diabetic complications (28% in patients with LSM < 7.0/6.2 kPa vs. 50% in patients with LSM ≥ 7.0/6.2 kPa, *p* < 0.001), including distal symmetric polyneuropathy (3% in patients with LSM < 7.0/6.2 kPa vs. 14% in patients with LSM ≥ 7.0/6.2 kPa, *p* < 0.05) [46]. Accumulating evidence also suggests the existence of an association between hepatic steatosis (as detected by imaging techniques) and cardiac autonomic dysfunction in patients with and without T2DM [49,50]. For instance, in a recent cross-sectional study including 173 individuals with T2DM and 183 age- and sex-matched nondiabetic controls from the Cooperative Health Research in South Tyrol (CHRIS) study, Targher et al. reported that individuals with T2DM and NAFLD (on ultrasonography) and individuals with NAFLD alone, but not those with T2DM alone, had an increased risk of cardiac sympathetic/parasympathetic imbalance (as assessed by low- to high-frequency power ratio and other heart rate variability measures obtained by a 20 min resting electrocardiogram), when compared with those without NAFLD and T2DM [50].

However, although this evidence is interesting, additional research is needed to corroborate these findings in larger populations and, more willingly, in longitudinal studies.

### 3.3. Diabetic Retinopathy

Some cross-sectional studies have investigated the relationship between NAFLD (as detected by imaging techniques) and the risk of prevalent diabetic retinopathy in patients with T2DM, reporting inconsistent results [44,51]. In this regard, a 2021 meta-analysis of nine cross-sectional studies for a total of 7170 patients with T2DM (57% with NAFLD on ultrasonography) reported no association between NAFLD and risk of prevalent diabetic retinopathy (random-effects odds ratio 0.94, 95% confidence interval 0.51–1.71; I^2^ = 96%) [52]. In addition, in that meta-analysis, subgroup analyses suggested that in China, Korea and Iran, T2DM patients with NAFLD had a decreased risk of diabetic retinopathy when compared with those without NAFLD, whereas in Italy and India, T2DM patients with NAFLD had an increased risk [52]. As suggested by the authors of that meta-analysis, the aforementioned results should be interpreted with caution, because of the high heterogeneity observed and the differences in the results seen across various countries. Hence, additional research is needed to better explore this issue [52].

## 4. Risk of Macrovascular Complications

### 4.1. Sub-Clinical Myocardial Remodelling and Dysfunction, Heart Valve Diseases and Cardiac Arrhythmias

A large body of evidence now supports the existence of a strong and independent association between NAFLD and sub-clinical myocardial remodelling and dysfunction, heart valve diseases (i.e., aortic-valve sclerosis and mitral annulus calcification) and cardiac arrhythmias (mainly atrial fibrillation) in patients with and without T2DM (Table 1) [13,22,23,53,54,55,56]. For instance, in a cross-sectional study involving 222 outpatients with T2DM (~70% with NAFLD on ultrasonography), our research group showed that NAFLD was associated with increased risk of left ventricular diastolic dysfunction (as evaluated by trans-thoracic echocardiography), independent of established CVD risk factors, diabetes-related covariates and other confounders [57]. Some recent observational studies using biopsy or vibration-controlled transient elastography (by FibroScan^®^) also observed a graded relationship between functional and structural myocardial abnormalities and NAFLD severity in patients with and without T2DM [22]. A 2019 meta-analysis of 16 observational studies further confirmed that NAFLD (as detected by imaging techniques or liver biopsy) was independently associated with many functional and structural myocardial abnormalities, including higher left ventricle mass, higher left ventricular end diastolic diameter, higher left atrium diameter and the ratio between left atrial volume and body surface area, higher posterior wall and septum thickness, lower E/A wave ratio, higher E/E′ ratio, longer deceleration time and longer relaxation time [58]. Interestingly, recent observational studies also indicated that NAFLD (as detected by ultrasonography) was associated with a reduction in global longitudinal strain, which is a relatively novel echocardiographic parameter strongly associated with adverse cardiovascular outcomes [59,60,61].

Relating to heart valve calcifications, some cross-sectional studies have shown an independent association between NAFLD and risk of aortic valve sclerosis (AVS) and mitral annulus calcification (MAC) in patients with and without T2DM [22,62]. For instance, in a study involving nearly 250 consecutive outpatients with T2DM (~70% with NAFLD on ultrasonography), our research group documented that NAFLD was strongly associated with cardiac calcifications in both the aortic and mitral valves, even after adjustment for established CVD risk factors, diabetes-related covariates and other confounders [62]. These findings may be clinically relevant, as functional and structural myocardial abnormalities and AVS/MAC are strongly associated with all-cause and cardiovascular mortality in patients with and without T2DM [63].

Relating to cardiac arrhythmias, several observational studies and some meta-analyses [64,65,66,67] have documented that NAFLD (as detected by imaging techniques) is associated with prevalent and incident permanent atrial fibrillation (AF) in patients with and without T2DM (Table 1) [22]. Notably, AF is, at present, the most frequent cardiac arrhythmia observed day by day in clinical practice and, importantly, it is strongly linked to adverse cardiovascular outcomes [22]. In a recent meta-analysis of five observational studies for a total of roughly 240,000 adult individuals with and without T2DM, our research group documented that NAFLD (as detected by imaging techniques) was associated with higher prevalence and incidence of AF [66]. Interestingly, in a recent retrospective longitudinal study including 267 patients (33% with NAFLD as detected by ultrasonography and 17% with T2DM at baseline) undergoing AF ablation, Donnellan et al. reported that NAFLD was associated with increased arrhythmia recurrence rates following AF ablation, during a mean follow-up of nearly 2.5 years [68]. Other observational studies and meta-analyses, also enrolling T2DM patients, have reported that NAFLD (as detected by ultrasonography) was associated with an increased risk of prolonged QTc, ventricular arrhythmias or conduction defects, independent of established cardiovascular risk factors, diabetes-related covariates and other confounders [22,67,69,70,71,72]. Interestingly, in a 2021 meta-analysis of 19 observational studies, Gong et al. confirmed that NAFLD (as detected by indirect markers of steatosis or imaging techniques) was independently associated with higher risks of prolonged QT interval (random-effects odds ratio 2.86, 95% confidence interval 1.64–4.99), premature atrial/ventricular contraction (random-effects odds ratio 2.53, 95% confidence interval 1.70–3.78) and heart block (random-effects odds ratio 2.65, 95% confidence interval 1.88–3.72) [67]. These data are clinically relevant, because NAFLD-related cardiac arrhythmias complications might contribute to explaining, at least in part, the increased risk of fatal and non-fatal CVD events observed in NAFLD patients.

### 4.2. Fatal and Non-Fatal Cardiovascular Events

Over the last few decades, it has become increasingly evident that the leading cause of death in NAFLD patients is CVD [22,23,73,74,75]. In this regard, using data from the National Vital Statistics System multiple-cause mortality data (2007–2016), Paik et al. reported that CVD was the main cause of death among US patients with NAFLD, as detected by ICD codes [74]. In a meta-analysis of 45 observational studies for a total of approximately 8 million individuals followed up to 13 years, Younossi et al. also estimated that the pooled CVD-specific mortality rate among NAFLD patients with or without T2DM was nearly 5 per 1000 person-years [3]. Several longitudinal studies and some meta-analyses confirmed that patients with NAFLD (as detected by imaging techniques, ICD codes or liver biopsy) have an increased risk of developing fatal and non-fatal CVD events, even after adjustment for several traditional CVD risk factors, diabetes-related variables, specific medications and other potential confounders (Table 1) [22,23,54,55,56,76,77,78,79]. In a 2021 meta-analysis of 36 longitudinal studies for a total of 5,802,226 adults and 99,668 incident cases of fatal and non-fatal CVD events over a median follow-up of 6.5 years, our research group reported that NAFLD (as detected by imaging techniques, ICD codes or liver biopsy) was associated with a 45% increased risk of fatal or non-fatal CVD events, independent of age, sex, body mass index, waist circumference, presence of T2DM and other cardiovascular risk factors (random-effects hazard ratio 1.45, 95% confidence interval 1.31–1.61; I^2^ = 86.2%) [14]. Such risk further increased in patients with severe forms of NAFLD, especially those with advanced fibrosis [14]. Another 2021 meta-analysis confirmed that NAFLD (as detected by imaging techniques, ICD codes or liver biopsy) was independently associated with increased risk of myocardial infarction (random-effects odds ratio 1.66, 95% confidence interval 1.39–1.99), ischemic stroke (random-effects odds ratio 1.41, 95% confidence interval 1.29–1.55) and heart failure (random-effects odds ratio 1.62, 95% confidence interval 1.43–1.84) [26]. In this regard, it is important to underline that the magnitude of cardiovascular risk is strongly related to the severity of NAFLD [25,80,81,82]. For instance, in a nationwide, matched cohort study of 10,568 Swedish individuals with biopsy-confirmed NAFLD (11% with T2DM at baseline) who were followed for a median period of 14 years, Simon et al. reported that, when compared to 49,925 adults of the general population (3% with established T2DM at baseline), mortality rates from CVD were significantly elevated in patients with simple steatosis (adjusted-hazard ratio 1.25, 95% confidence interval 1.16–1.35), and that these risks progressively increased in patients with NASH without fibrosis (adjusted-hazard ratio 1.66, 95% confidence interval 1.38–2.01), in those with non-cirrhotic fibrosis (adjusted-hazard ratio 1.40, 95% confidence interval 1.17–1.69) and also in those with cirrhosis (adjusted-hazard ratio 2.11, 95% confidence interval 1.63–2.73) [80]. Similar findings were also documented in cohorts involving NAFLD patients with T2DM [22,23,53,54,55,56].

To date, data regarding whether the improvement of NAFLD may reduce the incidence of cardiovascular complications are scarce. Although some retrospective studies enrolling Asian adults without pre-existing CVD have reported that the improvement or resolution of NAFLD (on ultrasonography) could be associated with a reduced risk of (carotid) atherosclerotic development in patients with and without T2DM [56,83], we believe that additional information on this issue is needed. In addition, it is important to underline that current evidence also indicates that histologic resolution of NASH could be associated with beneficial changes in risk factors for CVD [56,83], thus suggesting a potential favorable effect on cardiac complications.

Lastly, novel evidence also suggests that MAFLD criteria might identify patients with CVD better than NAFLD criteria [84].

## 5. CVD Risk Assessment in Patients with NAFLD

Based on the aforementioned evidence, the EASL-EASO-EASD and American Association for the Study of Liver Diseases (AASLD) practice guidelines for diagnosing and managing NAFLD now recommend a CVD risk assessment in all patients with NAFLD [1,2]. In this context, as suggested by several experts in the field [13], a potential comprehensive CVD risk assessment may include (Table 2): (a) evaluation of coexisting risk factors (such as a prior history of CVD, family history of premature CVDs or T2DM, cigarette smoking, presence of T2DM, dyslipidemia, hypertension, obesity, metabolic syndrome, chronic kidney disease and erectile dysfunction), (b) physical examination (such as body weight, height, body mass index, waist circumference, blood pressure, arterial bruits and pulse examination), (c) laboratory tests (such as blood count, lipid profile, fasting plasma glucose, HbA1c, serum creatinine, transaminases, albumin, urinalysis, albuminuria) and (d) cardiovascular examination tests (such as resting electrocardiogram, carotid artery ultrasonography, and exercise stress test if coexisting CVD, CKD, T2DM or >2 CVD risk factors). In addition, the current evidence on this topic also calls attention to a holistic approach in managing and treating NAFLD patients [75,85].

## 6. Conclusions

The aforementioned data support the concept that NAFLD is a “multisystemic” disease [13]. Indeed, NAFLD is not only associated with serious hepatic complications, but it is also linked with macro- and microvascular complications. Importantly and notably, at present, the main cause of death among NAFLD patients is CVD [14]. For this reason, a comprehensive CVD risk assessment is essential in these patients [1,2,13]. That said, information regarding the impact of histological improvement of NAFLD on CVD risk is still scarce and needs further research [56,83]. In spite of our knowledge about epidemiology, pathogenesis and natural history of NAFLD, no specific pharmacological therapies have until now been approved for such a disease [86]. Lifestyle change promoting weight loss and the correction of modifiable cardio-metabolic risk factors are still the cornerstone of the treatment in NAFLD patients [86]. However, over the last few decades, several potential agents have been tested to treat NAFLD and its advanced forms [86,87]. They encompass some glucose-lowering drugs (especially pioglitazone, glucagon-like peptide-1 [GLP-1] receptor agonists and sodium-glucose co-transporter-2 [SGLT-2] inhibitors) [87], bile and non-bile acid farnesoid X activated receptor (FXR) agonists, anti-oxidants (i.e., vitamin E), statins and others [86,88]. In this regard, for instance, in a 2022 systematic review of randomised controlled trials testing the efficacy of peroxisome proliferator-activated receptor (PPAR) agonists, GLP-1 receptor agonists and SGLT-2 inhibitors for treating NAFLD in adults with or without type 2 diabetes, our research group found that pioglitazone (a PPAR-γ agonist), lanifibranor (a pan-PPAR agonist) and GLP1-R agonists (e.g., liraglutide and semaglutide) are able to obtain the resolution of NASH without worsening of fibrosis, whereas SGLT-2 inhibitors (e.g., empagliflozin and dapagliflozin) are able to reduce liver fat content, as detected by magnetic resonance-based techniques [87]. Given the strong relationship between NAFLD and macro- and microvascular complications, it is possible to speculate that these agents may exert a beneficial effect not only on the hepatic disease, but also in reducing the risk of developing cardiovascular and renal diseases [25,86,87,88]. However, herein it is important to note that pioglitazone is contraindicated in patients with symptomatic heart failure or in patients with a high risk of heart failure [25]. Seeing the multiple pathways implicated in the pathogenesis of NAFLD and its complications, as well as the single response from single-agent therapies across RCTs available so far, it is also reasonable to hypothesize that the combination of different therapies (e.g., GLP-1 receptor agonists *plus* SGLT-2 inhibitors) will be more appropriate for treating NAFLD patients [86,87,89]. In this context, as suggested by several experts in the field, a holistic approach in managing and treating NAFLD patients seems to be fundamental [75,85].

## Figures and Tables

**Table 1:** Main meta-analyses of observational studies assessing the relationship between NAFLD and macro- and microvascular complications in patients with and without type 2 diabetes.

Author, Ref.	Main Study Characteristics	Main Results
**Fatal and non-fatal cardiovascular events**		
Targher G et al. *J. Hepatol.* 2016; 65: 589–600.	16 observational studies were included for a total of 34,043 individuals with and without T2DM	NAFLD was associated with an increased risk of fatal and/or non-fatal CVD (random-effects odds ratio 1.64, 95% confidence interval 1.26–2.13). Patients with more severe forms of NAFLD were also more likely to develop fatal and non-fatal CVD events (random-effects odds ratio 2.58; 95% confidence interval 1.78–3.75)
Morrison AE et al. *Liver Int.* 2019; 39: 557–567.	13 observational studies were included	NAFLD was not associated with an increased risk of CVD (random-effects risks ratio 1.48, 95% confidence interval 0.96–2.29)
Liu Y et al. *Sci Rep.* 2019; 9: 11124	14 observational studies were included for a total of 498,501 individuals with and without T2DM	NAFLD was associated with an increased risk of all-cause mortality (random-effects hazard ratio 1.34; 95% confidence interval 1.17–1.54), but not with an increased risk of CVD (random-effects hazard ratio 1.13; 95% confidence interval 0.92–1.38)
Mantovani A et al. *Lancet Gastroenterol Hepatol.* 2021; 6: 903–913	36 longitudinal studies were included for a total of 5,802,226 middle-aged individuals with and without T2DM	NAFLD was associated with an increased risk of fatal or non-fatal CVD events (random-effects hazard ratio 1.45, 95% confidence interval 1.31–1.61). This risk increased progressively across the severity of NAFLD, especially the stage of fibrosis (random-effects hazard ratio 2.50, 95% confidence interval 1.68–3.72)
Alon L et al. *Eur J Prev Cardiol.* 2021 Dec 22: zwab212. doi: 10.1093/eurjpc/zwab212.	20 observational studies were included	NAFLD was associated with an increased risk of myocardial infarction (random-effects odds ratio 1.66, 95% confidence interval 1.39–1.99), ischemic stroke (random-effects odds ratio 1.41, 95% confidence interval 1.29–1.55), atrial fibrillation (random-effects odds ratio 1.27, 95% confidence interval 1.18–1.37), and heart failure (random-effects odds ratio 1.62, 95% confidence interval 1.43–1.84)
**Cardiac function and structure**		
Borges-Canha M et al. *Endocrine* 2019; 66: 467–476.	16 observational studies were included	NAFLD was associated with increased risk of (a) higher left ventricle mass and ratios between left ventricle mass and both height and body surface area; (b) higher left ventricular end diastolic diameter; (c) higher left atrium diameter and ratio between left atrial volume and body surface area; (d) higher posterior wall and septum thickness; (e) lower E/A wave ratio; (f) higher E/E′ ratio; (g) longer deceleration time and (h) longer relaxation time
**Cardiac arrhythmias**		
Minhas AM et al. *Cureus* 2017; 9: e1142.	3 observational studies were included for a total of 1,044 with NAFLD and 1,016 without NAFLD	Patients with NAFLD had a higher risk of AF (random-effects odds ratio 2.47, 95% confidence interval 1.30–4.66)
Wijarnpreecha K et al. *Clin Res Hepatol Gastroenterol.* 2017; 41: 525–532	5 observational studies (2 cross-sectional ones and 3 cohort ones) were included for a total of 238,129 participants with and without T2DM	Patients with NAFLD had a higher risk of AF (random-effects risks ratio 2.06, 95% confidence interval 1.10–3.85)
Mantovani A. et al. *Liver Int.* 2019; 39: 758–769	9 observational studies (5 cross-sectional ones and 4 cohort ones) were included for a total of 364,919 individuals with and without T2DM	NAFLD was associated with an increased risk of prevalent AF (random-effects odds ratio 2.07, 95% confidence interval 1.38–3.10). Conversely, NAFLD was associated with increased risk of incident AF only in T2DM patients (random-effects hazard ratio 4.96, 95% confidence interval 1.42–17.3).
Gong H et al. *J. Int. Med. Res.* 2021; 49: 3000605211047074	19 observational studies were included for a total of 7,012,960 individuals with and without T2DM	NAFLD was associated with higher risks of AF (random-effects odds ratio 1.71, 95% confidence interval 1.14–2.57), prolonged QT interval (random-effects odds ratio 2.86, 95% confidence interval 1.64–4.99), premature atrial/ventricular contraction (random-effects odds ratio 2.53, 95% confidence interval 1.70–3.78) and heart block (random-effects odds ratio 2.65, 95% confidence interval 1.88–3.72)
**Chronic kidney disease (CKD)**		
Mantovani A et al. *Metabolism* 2018; 79: 64–76	9 observational studies were included for a total of 96,595 individuals with and without T2DM	NAFLD was associated with a higher risk of incident CKD (random-effects hazard ratio 1.37, 95% confidence interval 1.20–1.53). Patients with severe forms of NAFLD were more likely to develop incident CKD (random-effects hazard ratio 1.50, 95% confidence interval 1.25–1.74)
Mantovani A et al. *Gut* 2022; 71: 156–162	13 observational studies were included for a total of 1,222,032 individuals with and without T2DM	NAFLD was associated with an increased risk of incident CKD (random-effects hazard ratio 1.43, 95% confidence interval 1.33–1.54)

Abbreviations: AF, atrial fibrillation; CKD chronic kidney disease; CVD, cardiovascular disease; NAFLD, non-alcoholic fatty liver disease; T2DM, type 2 diabetes.

**Table 2:** Essential comprehensive cardiovascular risk assessment in patients with NAFLD.

Cardiovascular risk factors	History of CVD, family history of premature CVDs or T2DM, cigarette smoking, presence of T2DM, dyslipidemia, hypertension, obesity, CKD, erectile dysfunction (men), alcohol use
Physical examination	Weight, body mass index, waist circumference, blood pressure, pulse examination
Laboratory tests	Blood count (including hemoglobin and platelets), lipid profile, fasting glucose, HbA1c, serum creatinine, transaminases, albumin, albuminuria
Cardiovascular examination tests	Carotid artery ultrasonography, resting electrocardiogram, exercise stress test if coexisting CVD, CKD, T2DM or more than 2 CVD risk factors

This table is based on the review published by Byrne and Targher [13]. Abbreviations: CKD, chronic kidney disease; CVD, cardiovascular disease; T2DM type 2 diabetes.
